# Detection of a balanced translocation carrier through trophectoderm biopsy analysis: a case report

**DOI:** 10.1186/s13039-019-0444-2

**Published:** 2019-06-18

**Authors:** Olga Tšuiko, Tuuli Dmitrijeva, Katrin Kask, Pille Tammur, Neeme Tõnisson, Andres Salumets, Tatjana Jatsenko

**Affiliations:** 1 Competence Centre on Health Technologies, Tiigi 61b, 50410 Tartu, Estonia; 2 BioEximi OÜ, Sõle 23, 10614 Tallinn, Estonia; 3 Women’s Clinic, West-Tallinn Central Hospital, Sõle 23, 10614 Tallinn, Estonia; 4 Department of Clinical Genetics, United Laboratories, Tartu University Hospital, L. Puusepa 2, 51014 Tartu, Estonia; 5 Department of Clinical Genetics in Tallinn, United Laboratories, Tartu University Hospital, L. Puusepa 2, 51014 Tartu, Estonia; 6 Estonian Genome Center, University of Tartu, Riia 23b, 51010 Tartu, Estonia; 7 Institute of Bio- and Translational Medicine, University of Tartu, Ravila 19, 50411 Tartu, Estonia; 8 Department of Obstetrics and Gynaecology, University of Tartu, L. Puusepa 8, 50406 Tartu, Estonia; 9 Department of Obstetrics and Gynaecology, University of Helsinki and Helsinki University Hospital, Haartmaninkatu 2, 00029 Helsinki, Finland

**Keywords:** Balanced translocations, Preimplantation genetic testing, PGT-A, IVF, Next-generation sequencing

## Abstract

**Background:**

Balanced translocation carriers are burdened with fertility issues due to improper chromosome segregation in gametes, resulting in either implantation failure, miscarriage or birth of a child with chromosomal disorders. At the same time, these individuals are typically healthy with no signs of developmental problems, hence they often are unaware of their condition. Yet, because of difficulties in conceiving, balanced translocation carriers often turn to assisted reproduction, some of whom may also undergo preimplantation genetic testing for aneuploidy (PGT-A) to improve the likelihood of achieving a successful pregnancy.

**Case report:**

We describe a female patient, who pursued in vitro fertilization (IVF) treatment coupled with PGT-A following two consecutive miscarriages, unaware of her genetic condition. PGT-A was performed on blastocyst-stage embryos and the results of comprehensive chromosome screening from a first IVF cycle demonstrated reciprocal segmental aberrations on chromosome 7 and chromosome 10 in two out of four embryos. Due to distinct embryo profiles, the couple was then referred for genetic counselling and subsequent parental karyotyping revealed the presence of a previously undetected balanced translocation in the mother.

**Conclusions:**

These results confirm previous reports that genome-wide PGT-A can facilitate the identification of balanced translocation carriers in IVF patients, providing explanation for poor reproductive outcome and allowing adjustments in treatment strategies.

## Background

Any chromosomal aberrations in prospective parents, especially translocations, are often associated with reproductive failure, regardless of parental age. Balanced translocations can be categorized into reciprocal translocations, characterized by the exchange of genetic material between the two non-homologous chromosomes, and Robertsonian translocations, which occur as a result of a fusion of two acrocentric chromosomes (chromosomes 13, 14, 15, 21 or 22). Both reciprocal and Robertsonian translocations are one of the most common chromosomal abnormalities and the estimated frequency of all balanced structural rearrangements in the general population is 0.2–0.4% [1, 2]. Because there is very little or no loss of genetic material, balanced translocation carriers are phenotypically healthy, but they are at increased risk of having fertility issues. The defective chromosome segregation during meiosis can lead to unbalanced karyotype in the germ cells that can be transmitted to the embryo [3–5], resulting in either recurrent miscarriages or birth of a child with severe congenital disorders [6, 7]. Most translocation carriers are unaware of their condition, until parental karyotyping or genetic analysis of either aborted foetus or affected newborn is performed. Once diagnosed, more personalized treatment strategies are offered, and such couples can opt for the use of gamete donation or in vitro fertilization (IVF) treatment coupled with preimplantation genetic diagnosis (now also known as preimplantation genetic testing for structural rearrangements, or PGT-SR) to increase the chance of a successful pregnancy [8–11]. Following PGT-SR, only chromosomally balanced embryos are chosen for transfer based on the results of genetic analysis.

Here, we report a couple, who turned to assisted reproduction after experiencing two consecutive pregnancy losses. The couple chose to undergo preimplantation genetic testing for aneuploidy (PGT-A) to increase the likelihood of pregnancy per embryo transfer and reduce the risk of miscarriage. Comprehensive chromosome screening (CCS) revealed distinct copy-number changes on chromosome 7 and chromosome 10 in trophectoderm biopsies of two IVF embryos, which subsequently led to retrospective identification of a balanced translocation in the mother. In total, the patient underwent two IVF/PGT-A cycles and in the second cycle a pregnancy was established following the transfer of an euploid embryo, resulting in a birth of a healthy baby.

## Case presentation

### Material and methods

#### Patients

We report a 26-year-old female and her 28-year-old healthy male partner, who experienced difficulties in becoming pregnant since 2015. Female patient had a regular menstrual cycle, but was previously diagnosed with endometriosis in 2012 following laparoscopy, for which she received treatment with goserelin acetate implant (Zoladex®). In January and October 2016, the couple experienced two first trimester miscarriages after natural conception at 5/6 weeks (gestational sac and yolk sac were visible by obstetric ultrasonography) and at 4/5 weeks (only gestational sac was visible) of gestation, respectively. The couple then turned to assisted reproduction in 2017 due to fertility issues. Because of history of endometriosis, the female patient underwent laparoscopy again in April 2017, but no endometriotic lesions were found and fallopian tubes were patent. The female patient was then followed up for multiple cycles for the presence of a dominant follicle. In addition, she was administered with alpha chorionic gonadotropin (Ovitrelle®) and dihydrogesterone (Duphaston®) but failed to conceive. In September 2017, the couple enrolled into IVF/PGT-A program at fertility clinic at West-Tallinn Central Hospital for elective embryo transfer to assist in achieving a successful pregnancy. An informed consent was also obtained, allowing to use supernumerary/affected embryos for research purposes.

#### IVF treatment and embryo biopsy

Controlled ovarian stimulation was performed using recombinant follicle-stimulating hormone, followed by a gonadotropin-releasing hormone (GnRH) antagonist protocol. Final oocyte maturation was triggered by human chorionic gonadotropin administration 36–38 h prior to oocyte retrieval. In total 19 oocytes have been retrieved and all of them were fertilized by conventional IVF. The presumed zygotes were then cultured in a SAGE-1 single step media (Origio, Denmark) until day 5 blastocyst stage. Subsequent embryo morphological evaluation was performed according to the criteria set by Gardner and Schoolcraft [12]. Trophectoderm (TE) biopsy was performed on four embryos that reached the blastocyst stage using RI Saturn 5 Active™ Laser and on average 5–10 cells were aspirated per embryo. Following TE biopsy, all blastocysts were vitrified using MediCult Vitrification Cooling medias (Origio).

#### Comprehensive chromosome screening

For PGT-A, commercially available VeriSeq PGS kit (Illumina Inc., USA) was used for next-generation sequencing (NGS)-based aneuploidy screening. Briefly, TE biopsies were whole-genome amplified (WGA) according to ligation-mediated PCR-based SurePlex protocol (Illumina Inc., USA). The quality of WGA products was controlled on 1.5% agarose gel and the amount of amplified material was quantified by Qubit dsDNA HS Assay kit (Thermo Fisher Scientific, USA). Next, successfully amplified samples were used for library preparation, according to the manufacturer’s VeriSeq PGS kit protocol, and were sequenced on the Illumina MiSeq system. Subsequent CCS was performed using Illumina BlueFuse Multi v4.3 software with an embedded aneuploidy calling algorithm. Based on TE biopsy results, embryo classification was performed according to Preimplantation Genetic Diagnosis International Society (PGDIS) guidelines and recommendations for embryo prioritization (PGDIS, 2016).

#### Cytogenetic karyotyping

For blood cell karyotyping, conventional GTG-banding technique (G-bands by trypsin using Giemsa; band level 550) was used for staining metaphase chromosomes from peripheral blood lymphocytes. Chromosome aberrations were classified according to the International System for Human Cytogenetic Nomenclature (ISCN2016).

## Results

**Fig. 1:**
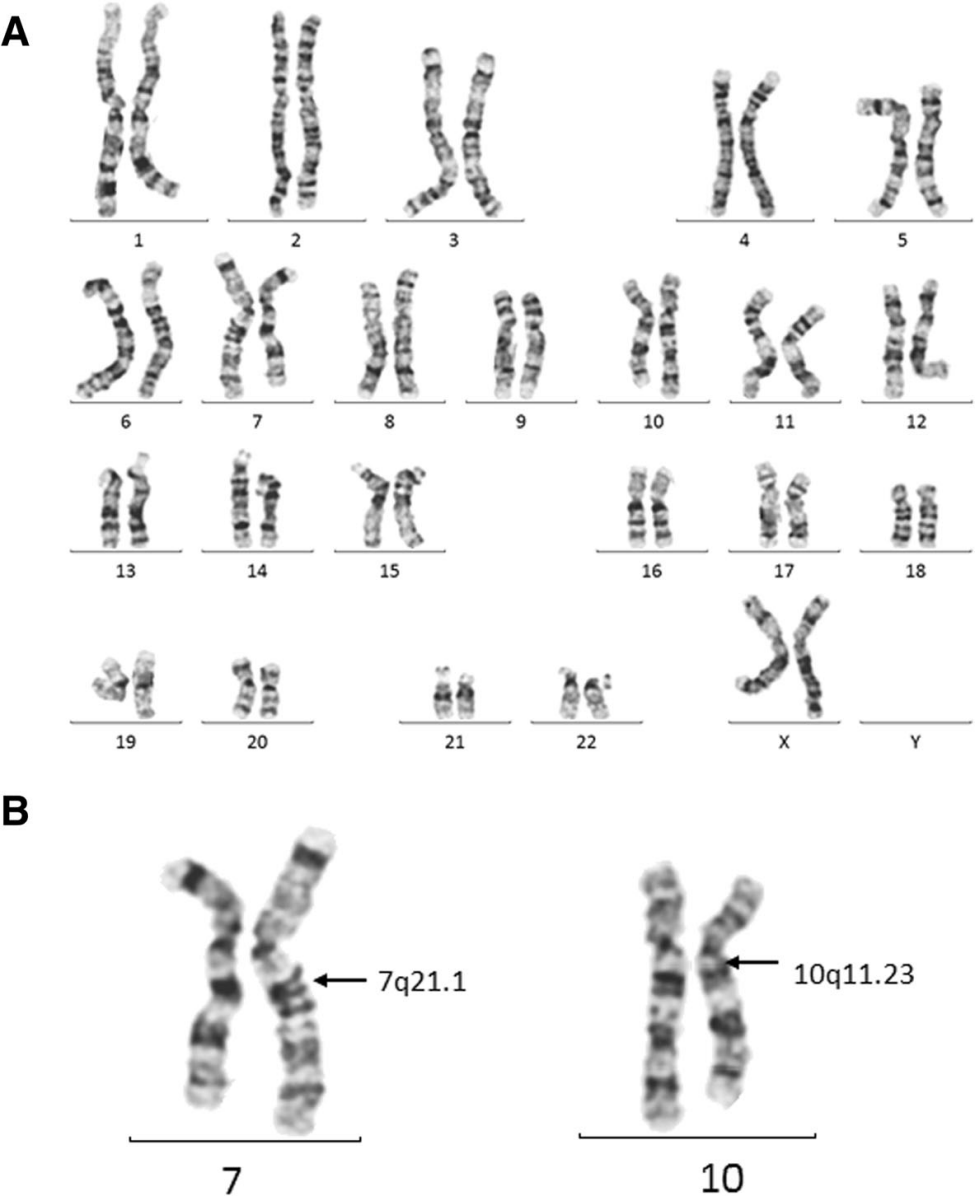
Maternal karyotype. Chromosome banding retrospectively revealed a translocation 46,XX,t(7;10)(q21.11;q11.23) in the mother. **a** Full maternal karyotype. **b** Detailed representation of translocated chromosomes 7 and 10. Arrows indicate breakpoints in rearranged chromosomes

**Fig. 2:**
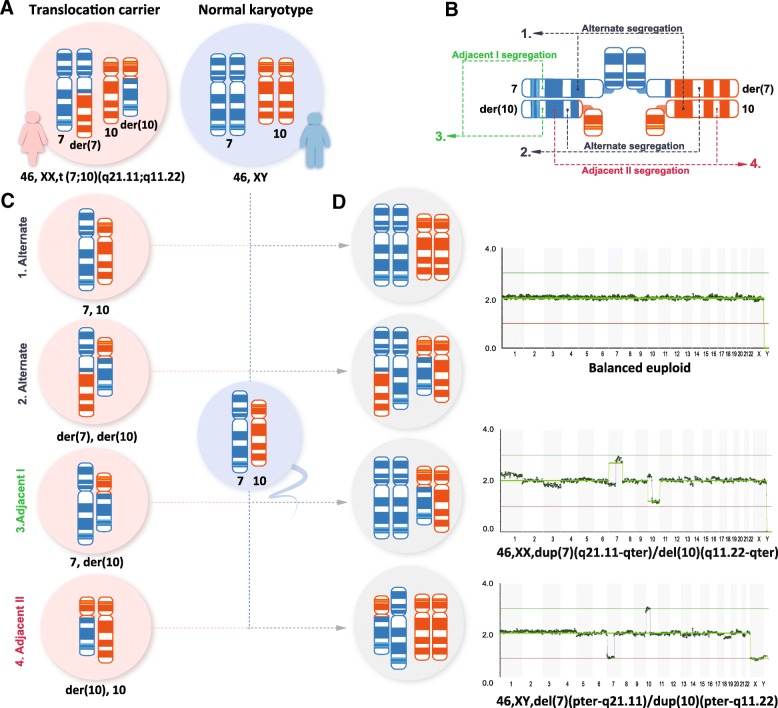
Identified meiotic segregation patterns and consequent embryo genomic profiles from reciprocal translocation carrier. Chromosomes, involved in balanced translocation in the mother (**a**), form a quadrivalent structure during meiotic pairing (**b**). Upon chromosome segregation, normal, balanced and unbalanced oocytes were generated as a result alternate segregation, adjacent I and adjacent II segregation (**c**). Upon fertilization, these oocytes give rise to embryos with or without genomic imbalances, as was identified by PGT-A (**d**)

The initial PGT-A analysis was performed on four TE biopsies, from which one embryo was predicted to be euploid (Embryo 2); two had recurrent segmental aberrations, involving distal regions of chromosomes 7 and 10 (Embryo 3 and Embryo 4); and one (Embryo 1) had a chaotic profile, characterized by multiple chromosome gains and losses (Table 1). Because of the distinct nature of detected segmental aberrations, which is common to translocation carriers, the couple was referred to genetic counselling, and parental karyotyping confirmed the presence of a reciprocal translocation in the mother (Fig. 1). As translocation carriers exhibit alternations in meiosis, segregation of quadrivalents during the meiotic division can produce germ cells with normal and balanced translocated karyotypes, and/or cells with unbalanced karyotype, according to adjacent I or adjacent II modes or 3:1 chromosomal segregation [13]. Such meiotic chromosome segregation patterns have also been previously observed in single sperm cells, derived from male translocation carriers [5], but depending on chromosomes involved, the overall proportion of different meiotic segregation modes can vary between male and female carriers [3]. In our case, the data indicated that Embryo 3 and 4 have inherited one normal and one derivative chromosome from the mother via adjacent II and adjacent I segregation, respectively (Fig. 2). Due to the diagnosis of a balanced translocation in the mother, the only euploid embryo from the first IVF cycle was re-classified as either 46,XX or 46,XX,t(7;10)(q21.11;q11.23) and transferred, but without resulting in pregnancy. Because most reciprocal translocations have various segregation modes, depending on chromosomes involved, localization of breakpoints and translocated segment sizes and carrier gender [14, 15], empirical data on the risks for viable unbalanced offspring can be lacking, and for each individual couple risk estimation is based on the detected balanced translocation and family history. In the current case, no further counselling was requested by the couple after the diagnosis of a balanced translocation in the mother, and the female patient continued with IVF treatment and embryo testing. In the second cycle, following the same stimulation protocol, three embryos were biopsied and analysed (Table 1). Although Embryo 6 presented with monosomy 6 and multiple aneuploidies, segmental rearrangements of chromosome 7 and 10 were also detected, signifying the defective meiotic segregation patterns of translocated chromosomes. Based on the outcome of embryo chromosome screening from the second IVF cycle, Embryo 7 with a balanced 46,XX/46,XX,t(7;10)(q21.11;q11.23) karyotype was chosen for transfer, resulting in a singleton pregnancy and live birth in December 2018.

**Table 1:** Results of preimplantation genetic testing for aneuploidy (PGT-A)

IVF cycle	Embryo ID	Embryo Grade	PGT-A result
1	1	5AB	Chaotic with multiple chromosome gains and losses
	**2**	5AB	46,XX / 46,XX,t(7;10)(q21.11;q11.23)^a^
	3	4BB	46,XY,del (7)(pter-q21.11)/dup (10)(pter-q11.23)
	4	5AB	46,XX,dup (7)(q21.11-qter)/del (10)(q11.23-qter)
2	5	4AB	46,XX,del (3)(q26.1-q29)
	6	4AB	45,XY,-6, del (7)(pter-q21.11)/dup (10)(pter-q11.23); multiple mosaic aneuploidies
	**7**	5BB	46,XX / 46,XX,t(7;10)(q21.11;q11.23)^a^

^a^Euploid embryos were re-classified after the detection of balanced translocation in the mother

## Discussion

In this case report, we described a retrospective identification of a balanced reciprocal translocation carrier, based on the embryonic profiles obtained after PGT-A. This discovery together with diagnosed endometriosis provided an extensive aetiology on reproductive failure in the female patient. We have also corroborated previous reports, indicating that PGT-A can identify patients at risk of carrying balanced genomic rearrangements upon observations made in the embryos [16–18].

The frequency of adverse reproductive outcomes can reach up to 5% in translocation carriers, compared to < 1% in general population [19]. Even though subfertile couples, undergoing IVF treatment, have an increased prevalence of structural chromosomal rearrangements in comparison to a general population [20–25], routine prenatal karyotyping is not a part of the standard IVF work-up. Instead, selective karyotyping can be indicated for patients with recurrent implantation failure [25] or recurrent pregnancy loss (RPL) [26], which was generally defined as ≥3 consecutive miscarriages [27]. Because our patient had a history of endometriosis, which was considered as a confounding factor, and experienced two early consecutive pregnancy losses, she was not referred for genetic testing based on the genetic counselling guidelines in Estonia that adopted the previously existing RPL definition. However, there has been a significant debate regarding the definition of RPL and patient management, and recent ESHRE guidelines recommend that a diagnosis of RPL should be considered after ≥2 consecutive pregnancy losses [28]. Since miscarriage poses a tremendous psychological burden for any couple, especially to female patients, IVF/PGT-A option may still seem attractive, despite the controversy and high costs [29]. However, adequately adopted selective karyotyping prior to IVF, especially in patients with a history of miscarriages and/or in cases of severe male infertility, can hold additional clinical and financial benefits [26]. First, parental karyotyping can provide the genetic cause of infertility, as chromosomal abnormalities can lead to gametogenesis failure [30–32]. This information can be important to patients and can aid in patient management and informed decision making for best treatment options. Second, in case of identified structural rearrangements, such as balanced translocations, PGT-SR can be indicated to avoid the transfer of an affected embryo with unbalanced karyotype [8, 10]. Unlike PGT-A, PGT-SR involves diagnosis of inherited structural rearrangements in the embryo, thus it is more likely to be reimbursed by national healthcare systems.

Traditionally, PGT-SR has been performed using fluorescent in situ hybridization, but genome-wide screening via array comparative genomics hybridization or NGS can be performed. Although conceptually PGT-SR is different from PGT-A, comprehensive chromosome screening of the whole genome can have an additional diagnostic value, as it allows to detect other chromosomal imbalances, unrelated to parental translocation, that would normally be missed by targeted approaches. In our case, Embryo 5 had a balanced profile for chromosomes 7 and 10, but a full segmental deletion on chromosome 3 was also detected. Nowadays, NGS techniques with increased sensitivity and resolution are very rapidly implemented into the clinical practice for full genome screening, as they can also detect chromosomal mosaicism [33]. Although extensive knowledge about the effect of mosaic aneuploidies on pregnancy is lacking, this approach allows to perform embryo ranking and subsequent transfer of the most viable embryos first, based on their genomic content and degree of mosaicism [34]. However, chromosomal mosaicism at blastocyst stage still represents a major clinical challenge in patient management, especially when only mosaic embryos are available for transfer.

In conclusion, we reported a case of retrospective balanced translocation carrier identification via blastocyst biopsy analysis. Given that conventional karyotyping is not routinely performed in fertility treatment, the increased use of PGT-A will likely facilitate the detection of undiagnosed balanced translocation carriers among IVF patients.

## Data Availability

Data generated and analysed during the study are included in this published case report. Full datasets used in the current study are available from the corresponding author on reasonable request.

## Abbreviations

CCS: Comprehensive chromosome screening
IVF: In vitro fertilization
NGS: Next-generation sequencing
PGT-A: Preimplantation genetic testing for aneuploidy
PGT-SR: Preimplantation genetic testing for structural rearrangements
RPL: Recurrent pregnancy loss
TE: Trophectoderm
WGA: Whole genome amplification
